# Influence of APOE Genotype on Hippocampal Atrophy over Time - An N=1925 Surface-Based ADNI Study

**DOI:** 10.1371/journal.pone.0152901

**Published:** 2016-04-11

**Authors:** Bolun Li, Jie Shi, Boris A. Gutman, Leslie C. Baxter, Paul M. Thompson, Richard J. Caselli, Yalin Wang, Alzheimer’s Disease Neuroimaging Initiative

**Affiliations:** 1 School of Computing, Informatics, and Decision Systems Engineering, Arizona State University, Tempe, Arizona, United Stated of America; 2 Imaging Genetics Center, Institute for Neuroimaging and Informatics, University of Southern California, Marina del Rey, California, United Stated of America; 3 Human Brain Imaging Laboratory, Barrow Neurological Institute, Phoenix, Arizona, United Stated of America; 4 Department of Neurology, Mayo Clinic Arizona, Scottsdale, Arizona, United Stated of America; Duke University, UNITED STATES

## Abstract

The apolipoprotein E (APOE) e4 genotype is a powerful risk factor for late-onset Alzheimer’s disease (AD). In the Alzheimer’s Disease Neuroimaging Initiative (ADNI) cohort, we previously reported significant baseline structural differences in APOE e4 carriers relative to non-carriers, involving the left hippocampus more than the right—a difference more pronounced in e4 homozygotes than heterozygotes. We now examine the longitudinal effects of APOE genotype on hippocampal morphometry at 6-, 12- and 24-months, in the ADNI cohort. We employed a new automated surface registration system based on conformal geometry and tensor-based morphometry. Among different hippocampal surfaces, we computed high-order correspondences, using a novel inverse-consistent surface-based fluid registration method and multivariate statistics consisting of multivariate tensor-based morphometry (mTBM) and radial distance. At each time point, using Hotelling’s *T*^2^ test, we found significant morphological deformation in APOE e4 carriers relative to non-carriers in the full cohort as well as in the non-demented (pooled MCI and control) subjects at each follow-up interval. In the complete ADNI cohort, we found greater atrophy of the left hippocampus than the right, and this asymmetry was more pronounced in e4 homozygotes than heterozygotes. These findings, combined with our earlier investigations, demonstrate an e4 dose effect on accelerated hippocampal atrophy, and support the enrichment of prevention trial cohorts with e4 carriers.

## Introduction

Alzheimer’s disease (AD) is the most common cause of dementia, accounting for 60–80% of cases [1, 2]. Effective presymptomatic diagnosis and treatment of AD could have enormous public health benefits. The underlying pathology of AD precedes the onset of cognitive symptoms by many years, and efforts are underway to find reliable preclinical diagnostic biomarkers. The discovery of APOE as the most prevalent known genetic risk factor for AD [3, 4] has made it possible to study large numbers of genetically at-risk individuals before the onset of symptomatic memory impairment. This led to the concept of *preclinical AD* [5], which has now been validated in autopsy studies of non-demented elderly subjects with neuropathological evidence of AD [6–10], brain imaging studies [11–19], amyloid detection [20], and neuropsychological studies [21, 22]. Surface-based subregional structure analysis may offer additional benefits [17, 23–26], such as better visualization and increased statistical power, especially when detecting subtle genetic effects. As the paradigm in experimental therapeutics shifts toward earlier intervention and prevention, enrichment of treatment cohorts with APOE e4 carriers may improve diagnostic accuracy and may make it faster to evaluate treatments for preclinical AD [27, 28].

Structural magnetic resonance imaging (MRI) measurements of regional and whole brain tissue shrinkage, together with fluorodeoxyglucose positron emission tomography (FDG PET) measures of decline in the regional cerebral metabolic rate for glucose (CMRgl), and PET measurements of fibrillar amyloid-β (Aβ) burden are among the best established imaging biomarkers for preclinical detection and tracking of AD [29]. In AD research, commonly-used structural MRI measures include whole-brain [30–32], entorhinal cortex [33], hippocampus [25, 34–38], and temporal lobe volumes [39], as well as ventricular enlargement [35, 38, 40, 41]. Reductions in hippocampal and entorhinal cortex volumes become apparent in the early stages of memory decline and may anticipate progression to MCI and AD [42, 43]. Hippocampal atrophy measures from structural MRI are widely used, but do not generally detect more subtle alterations in hippocampal morphometry that may provide even more sensitive detection of early change.

In our recent work [25], we automatically segmented and constructed hippocampal surfaces from the baseline MR images of 725 subjects with known APOE genotype—including 167 with sporadic AD, 354 with MCI, and 204 normal controls. We also built high-order correspondences between hippocampal surfaces and computed multivariate statistics consisting of multivariate tensor-based morphometry (mTBM) and radial distance. Using Hotelling’s *T*^*2*^ test, we found significant morphological deformation in APOE e4 carriers relative to non-carriers in the entire cohort as well as in the non-demented (pooled MCI and control) subjects, affecting the left hippocampus more than the right (left hippocampus has a greater dose effect of APOE e4 than the right), and this effect was more pronounced in e4 homozygotes than heterozygotes. We now extend our work to a large, longitudinal dataset of brain MR images (N = 1925) from the Alzheimer’s Disease Neuroimaging Initiative (ADNI) acquired at baseline, 6-month (N = 724), 12-month (N = 673) and 24-month (N = 533) intervals. We applied a novel surface mTBM system [40, 44–47] to create 3D maps of hippocampal atrophy associated with the APOE4 genotype. We hypothesized that (1) we would observe similar patterns of hippocampal deformation at each time points, as previously observed in the baseline study [25], and (2) the severity of hippocampal deformation and rate of decline would parallel APOE e4 gene dose.

## Subjects and Methods

### Subjects

Data used in this paper were obtained from the Alzheimer’s Disease Neuroimaging Initiative (ADNI) database (adni.loni.usc.edu). The ADNI was launched in 2003 by the National Institute on Aging (NIA), the National Institute of Biomedical Imaging and Bioengineering (NIBIB), the Food and Drug Administration (FDA), private pharmaceutical companies and non-profit organizations, as a $60 million, 5-year public-private partnership. The primary goal of ADNI has been to test whether serial magnetic reasonance imaging (MRI), positron emission tomography (PET), other biological markers, and clinical and neuropsychological assessment can be combined to measure the progression of mild cognitive impairment (MCI) and early Alzheimer’s disease (AD). Determination of sensitive and specific markers of very early AD progression is intended to aid researchers and clinicians to develop new treatments and monitor their effectiveness, as well as lessen the time and cost of clinical trials.

The Principal Investigator of this initiative is Michael W. Weiner, MD, VA Medical Center and University of California—San Francisco. ADNI is the result of efforts of many co-investigators from a broad range of academic institutions and private corporations, and subjects have been recruited from over 50 sites across the U.S. and Canada. The initial goal of ADNI was to recruit 800 subjects but ADNI has been followed by ADNI-GO and ADNI-2. To date these three protocols have recruited over 1500 adults, ages 55 to 90, to participate in the research, consisting of cognitively normal older individuals, people with early or late MCI, and people with early AD. The follow up duration of each group is specified in the protocols for ADNI-1, ADNI-2 and ADNIGO. Subjects originally recruited for ADNI-1 and ADNI-GO had the option to be followed in ADNI-2. For up-to-date information, see www.adni-info.org.

At the time of downloading (September 2011), the baseline dataset consisted of 843 adults, including 233 elderly healthy controls (CTL), 410 subjects with mild cognitive impairment (MCI) and 200 AD patients. The 6-month follow up cohort consisted of 738 adults, including 214 elderly healthy controls (CTL), 359 subjects with mild cognitive impairment (MCI) and 165 AD patients. The 12-month follow up cohort consisted of 685 adults, including 203 elderly healthy controls (CTL), 338 subjects with mild cognitive impairment (MCI) and 144 AD patients. The 24-month follow up cohort consisted of 543 adults, including 178 elderly healthy controls (CTL), 254 subjects with mild cognitive impairment (MCI) and 111 AD patients. All subjects underwent thorough clinical and cognitive assessment at the time of acquisition, including the Mini-Mental State Examination [48], Clinical Dementia Rating (CDR) [49], and Delayed Logical Memory Test [50]. APOE genotyping was performed on DNA samples obtained from subjects’ blood, using an APOE genotyping kit, as described in http://www.adni-info.org/Scientists/Pdfs/adniproceduresmanual12.pdf (also see http://www.adni-info.org for detailed information on blood sample collection, DNA preparation, and genotyping methods).

Participants were scanned with a standardized MRI protocol developed for this cohort [51]. We applied our hippocampal morphometry pipeline [25, 46] to reconstruct hippocampal meshes (detailed in Sec. 2.3). As a quality control, we manually checked all the constructed meshes. Similar to our prior work [25, 46], the exclusion criteria include: (1) failing the FIRST segmentation step probably due to the original images’ resolution or contrast issue; (2) wrong surface topologies, such as the generated hippocampal surfaces have handles. In 6-month data, we manually excluded 3 subjects from CTL group, 6 subjects from MCI group and 5 subjects from AD group with wrong surface topologies. Similarly, in 12-month data, we manually excluded 3 subjects from CTL group, 8 subjects from MCI group, and 1 subject from AD group. In 24-month data, we manually excluded 2 subjects from CTL group, 5 subjects from MCI group, and 3 subjects from AD group. As a result, a total of 1925 ADNI longitudinal brain MR scans, including 211 controls (with a mean age of 76.41), 353 MCI (mean age: 75.06), and 160 AD (mean age: 74.88) from the 6-month follow-up cohort, 200 controls (mean age: 76.38), 330 MCI (mean age: 74.82), and 143 AD (mean age: 75.63) from the 12-month follow-up cohort, 176 controls (mean age: 76.44), 249 MCI (mean age: 74.75), and 108 AD (mean age: 75.17) from the 24-month follow-up cohort, were analyzed in the study. Table 1 gives detailed demographic data information on the subjects.

**Table 1:** Table of Demographic Data by Diagnositic and Genotype Groups. Demographic data by diagnositic and genotype groups. N_6_, N_12_, and N_24_ indicate sample size of the 6-month, 12-month and 24-month follow up cohorts, respectively. The number of women in the samples is indicated in parentheses. Means are followed by standard deviations in parentheses for age and MMSE measures.

APOE genotype	CTL	MCI	AD	Total
**0 APOE e4 allele (e3/e3)**				
**N**_**6**_	115(52)	127(44)	43(21)	285(117)
**N**_**12**_	104(46)	120(41)	40(18)	264(105)
**N**_**24**_	98(46)	90(33)	28(13)	216(92)
**Age**	76.51(±4.91)	76.13(±7.53)	76.82(±8.55)	76.39(±6.75)
**MMSE**	29.10(±1.13)	26.54(±3.36)	20.79(±5.00)	26.77(±4.06)
**1 APOE e4 allele (e3/e4)**				
**N**_**6**_	43(21)	125(44)	61(25)	229(90)
**N**_**12**_	44(21)	117(41)	58(25)	219(87)
**N**_**24**_	40(19)	86(26)	45(18)	171(63)
**Age**	76.43(±4.42)	74.67(±6.65)	75.70(±6.06)	75.30(±6.13)
**MMSE**	28.79(±2.92)	25.41(±3.59)	21.25(±4.66)	24.96(±4.69)
**2 APOE e4 alleles (e4/e4)**				
**N**_**6**_	3(1)	41(18)	32(13)	76(32)
**N**_**12**_	4(2)	39(16)	28(10)	71(28)
**N**_**24**_	4(2)	31(12)	21(9)	56(23)
**Age**	73.36(±2.92)	71.82(±5.74)	72.07(±6.91)	72.00(±6.13)
**MMSE**	29.09(±1.58)	25.68(±3.31)	20.72(±5.23)	23.88(±4.92)
**Total**				
**N**_**6**_	161(74)	293(106)	136(59)	590(239)
**N**_**12**_	152(69)	276(98)	126(53)	554(220)
**N**_**24**_	142(67)	207(71)	94(40)	443(178)
**Age**	76.41(±4.76)	74.89(±7.07)	75.22(±7.31)	75.40(±6.58)
**MMSE**	29.01(±1.82)	25.94(±3.49)	20.99(±4.89)	25.71(±4.52)

In our study, following prior work [23, 25, 46, 52], at each time point we pooled both the subjects who are heterozygotes APOE e4 carriers (e3/e4) and homozygotes APOE e4 carriers (e4/e4) together to form the *APOE e4 carriers group* and correlated presence of the APOE e4 allele with hippocampal morphometry, both (1) in the entire sample and (2) in non-demented (pooled MCI and controls) subjects. Throughout the paper, we call these two populations as the *full ADNI cohort* and *non-demented cohort*, respectively.

### Processing Pipeline

**Fig 1:**
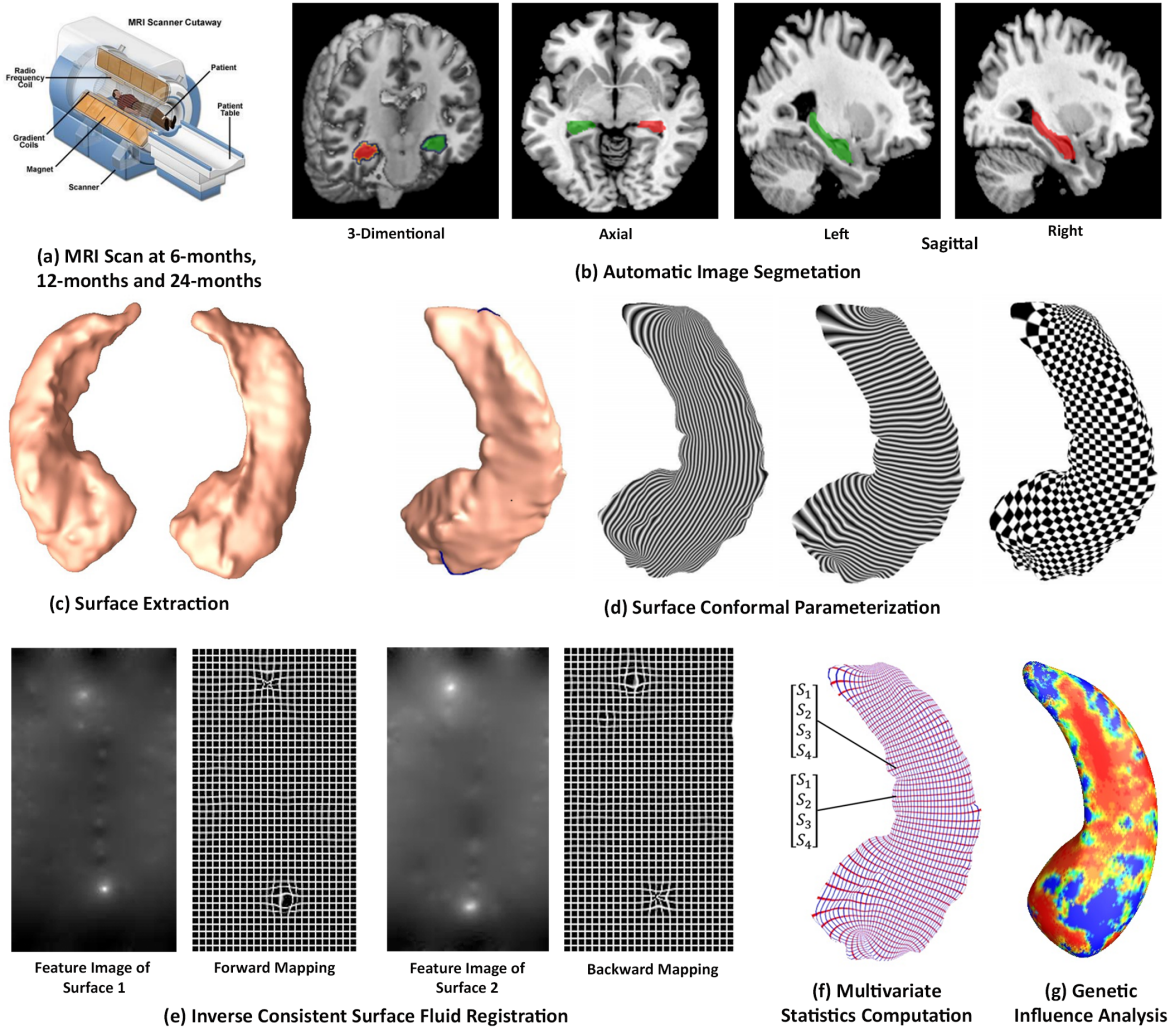
Overall Processing Sequence. (a): Longitudinal data (6-months, 12-months and 24-months) were obtained from the Alzheimer’s Disease Neuroimaging Initiative (ADNI) database; (b) automatic hippocampus segmentation with FIRST software [53]; (c) hippocamal surface reconstruction with marching cube method [58]; (d) hippocampal surface conformal parameterization with holomorphic 1-forms [54]; (e) inverse consistent surface fluid registration of hippocampal surfaces [46]; (f) multivariate statistics [44] consisting of mTBM and radial distance; (g) longitudinal genetic influence of APOE e4 allele on hippocampal morphometry.

Fig 1 summarizes the overall processing sequence. The original input data were the three-dimensional (3D) T1-weighted images from ADNI dataset (6-months, 12-months and 24-months), an example image is shown in Fig 1(a). First, we used the FIRST (FMRIB’s Integrated Registration and Segmentation Tool) software [53] to segment the original data and obtain the hippocampus substructure. The hippocampal surfaces were automatically reconstructed based on binary segmentation results [25, 46]. Second, we generated a conformal grid for each surface with the holomorphic 1-form basis [54]. With the help of conformal grid, we can compute the conformal representation as the “feature image” of a surface. Third, we registered the feature image of each surface in the dataset to a common template with an inverse consistent surface fluid registration algorithm. Finally, we studied the longitudinal differences between different groups with the mTBM statistics [44] together with the radial distance. The similar processing pipeline was used in several of our prior works [25, 40, 47, 55–57].

### Hippocampus Segmentation and Surface Modeling

In the process of segmentation, we used FIRST [53] to automatically process all T1-weighted MR images. FIRST is a model based subcortical structure segmentation and registration tool developed as part of the FSL library, which is written mainly by members of the Analysis Group, FMRIB, Oxford, UK. Within FIRST, we ran the *run_first_all* routine with default parameters tuned by FIRST as optimal for hippocampal segmentation. For now, we took three-phase image which contains the labels of the left and right hippocampi. The binary image of each side of hippocampus was obtained by a simple thresholding process. Fig 1(b) shows an example of segmented hippocampus substructure. Then hippocampal surfaces were constructed with the marching cubes algorithm [58]. After mesh refinement [25, 46], we obtained smooth surfaces that are suitable for generating conformal grids. Finally, with the help of global affine transformation with a nine-parameter (three parameters for translation, three parameters for rotation, and three parameters for scaling) matrix that was computed by FIRST, the smoothed meshes were aligned into the MNI standard space. Fig 1(c) shows a pair of reconstructed hippocampal surfaces.

### Conformal Grid Generation and Surface Conformal Representation

To facilitate hippocampal shape analysis, we generated a conformal grid on each surface, which is used as a canonical space for surface registration. On each hippocampal surface, we computed its conformal grid with holomorphic 1-form basis [44, 54]. Fig 1(d) shows an example hippocampal surface with its exact 1-form basis, conjugate 1-form basis and holomorphic 1-form basis [44, 54]. In the picture, the overlaid texture is used to demonstrate the computed 1-form bases. The checkboard texture is used to show the angle preserving property.

We adopted surface conformal representation [25, 46] to obtain surface geometric features for automated surface registration. It consists of the conformal factor and mean curvature, encoding both intrinsic surface structure and information on its 3D embedding. After we computed these two local features on each surface point, we computed their summation and then linearly scaled the dynamic range of the summation into [0, 255] to obtain a feature image for the surface.

### Hippocampal Surface Registration with Inverse-Consistent Surface Fluid Registration

For longitudinal morphometric analysis, we need to register each individual hippocampal surface to a common template surface. With surface conformal parameterization and conformal representation, we generalized the well-studied image fluid registration algorithm [59, 60] to general surfaces. Furthermore, most image registration algorithms in the literature are not symmetric, i.e., the correspondences established between the two images depend on which image is assigned as the deforming image and which is the non-deforming target image. An asymmetric algorithm can be problematic as it tends to penalize the expansion of image regions more than shrinkage [61]. Thus, in our system, we further extended the surface fluid registration method to an inverse-consistent framework [62]. The obtained surface registration is diffeomorphic. For details of our inverse-consistent surface fluid registration method, we refer to [46]. Fig 1(e) illustrates the surface inverse consistent fluid registration method.

### Surface Multivariate Morphometry Statistics

Our multivariate morphometry statistical analysis consists of mTBM [25, 40, 44, 46] and radial distance analysis [35, 63]. This combines complementary information from mTBM, which measures deformation within surfaces, and radial distance, which measures hippocampal size in terms of the surface normal direction.

As in our prior work [44], the mTBM was computed as a 3 × 1 vector consisting of the “Log-Euclidean metric” [64], computed as the matrix logarithm of the deformation tensor. mTBM statistics have been carefully studied in brain structure morphology analyses and they can detect signals more powerfully than more standard Jacobian matrix statistics [40, 45–47, 55]. Given the hippocampal tube-like shape, its atrophy and enlargement directly affect the distance from each surface point to its medial core (analogous to the center line in a tube). We call this distance the *radial distance* of a hippocampal surface. We formed the new multivariate surface morphometry statistic as a 4 × 1 vector consisting of the mTBM and radial distance (Fig 1(f)).

### Statistical Group Difference

To assess group differences with multivariate statistics, we applied Hotelling's *T*^2^ test [65–68] on sets of values of the new multivariate statistics. For each surface vertex, given two groups of *n*×4-dimensional vectors, *S*_*i*_, *i* = 1,2,…, *p*,*T*_*j*_, *j* = 1,2,…, *q*, we used the Mahalanobis distance *M* to measure the group mean difference,

$$M=\frac{N_{S}N_{T}}{N_{S}+N_{T}}{\left(\bar{S}-\bar{T}\right)}^{T}\Sigma^{-1}\left(\bar{S}-\bar{T}\right).$$

where *N*_*s*_ and *N*_*T*_ are the numbers of subjects in the two groups, $\overset{-}{S}$ and $\overset{-}{T}$ are the means of the two groups and Σ is the combined covariance matrix of the two groups [40, 44, 69].

Next, for each hippocampal surface point, we ran a permutation test with 10,000 random assignments of subjects to different groups to estimate the statistical significance of the areas with group difference in surface morphometry. We also used a pre-defined statistical threshold of *p* = 0.05 at each surface point to estimate the overall significance of the group difference maps by non-parametric permutation testing [70, 71]. In each case, the covariate (group membership) was permuted 10,000 times and a null distribution was developed for the area of the average surface with group difference statistics above the pre-defined threshold in the significance map. The *overall significance of the map* is defined as the probability of finding, by chance alone, a statistical map with at least as large a surface area beating the pre-defined statistical threshold of *p* = 0.05. The permutation test on the overall rejection areas is used to evaluate the significance of overall experimental results and correct the overall significant *p*-values for multiple comparisons. Fig 1(g) shows an example of the significance *p*-map with uncorrected *p*-values, which is used to visualize the surface regions with significant differences between groups.

## Results

Similar to our prior work [25], we mainly focused on studying the effects of APOE e4 genotype on hippocampal morphometry in two populations, (1) the full ADNI cohort; and (2) the non-demented cohort, i.e., people with MCI and normal control subjects. APOE e3 is the most prevalent allelic variant and considered the human wild type. In a gene-dose dependent pattern, APOE e4 increases and APOE e2 decreases susceptibility to “sporadic” and late onset AD [3, 4, 72–75]. APOE e4 is the focus of research in this paper. We took an approach similar to our prior work [23, 25], and excluded e2 carriers (with the result that a total of 60 e2 allele carriers were excluded from the study).

### Results in the Full ADNI Cohorts for Three Follow Up Intervals

**Fig 2:**
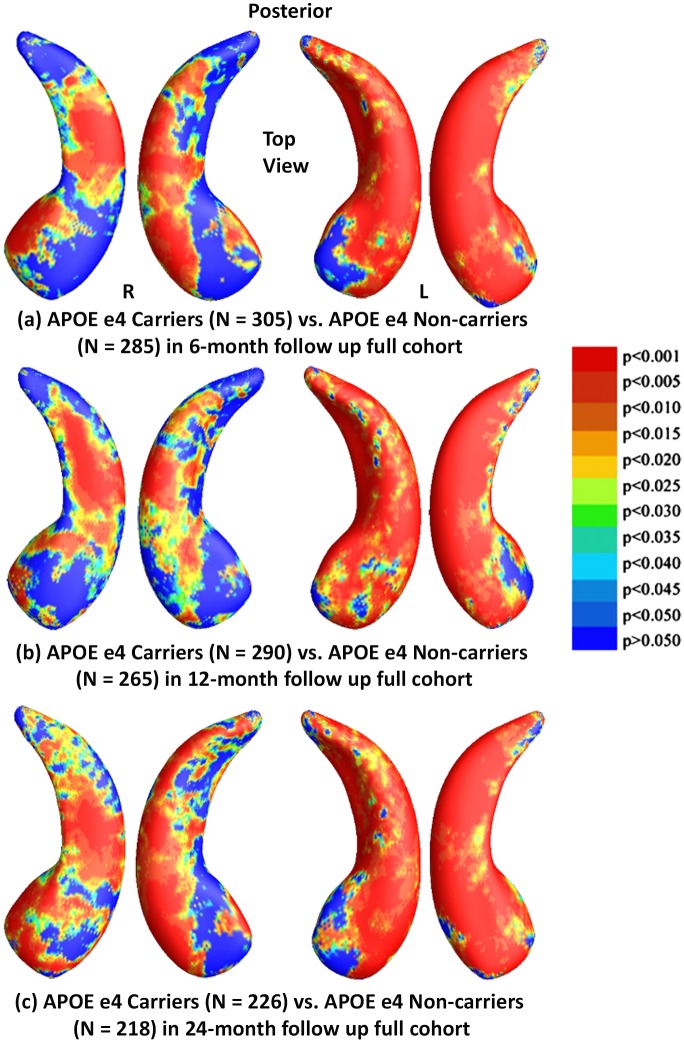
Shape Differences between Non-carriers and Carriers in Full ADNI. (a), (b) and (c): Illustration of local shape differences (*p*-values) between the APOE e4 carriers **(e3/e4 and e4/e4)** and non-carriers **(e3/e3)** in the full ADNI cohorts at 6-months, 12-months and 24-months respectively. Non-blue colours show vertices with statistical differences, at the nominal 0.05 level, uncorrected. The overall significance after multiple comparisons with permutation test is: (a) *p* < 0.0001, (b) *p* < 0.0001, (c) *p* < 0.0005.

To explore whether the presence of the APOE e4 allele was associated with greater hippocampal atrophy, we studied the effects of APOE e4 genotype in three follow up full ADNI cohorts. Fig 2 shows the statistical *p*-maps for comparisons between e4 carriers (e3/e4 and e4/e4) and e4 non-carriers (e3/e3) in these three different follow up intervals, specifically, (a) for 6-month follow up (N = 590, 285 non-carriers vs. 305 carriers), (b) for 12-month (N = 555, 265 non-carriers vs. 290 carriers), and (c) for 24-month (N = 444, 218 non-carriers vs. 226 carriers). Non-blue colours show vertices with statistical differences at the nominal 0.05 level, uncorrected for multiple comparisons. After correcting for multiple comparisons, the differences remained highly significant (*p*<0.0001 for 6-months and 12-months, *p*<0.0005 for 24-months).

**Fig 3:**
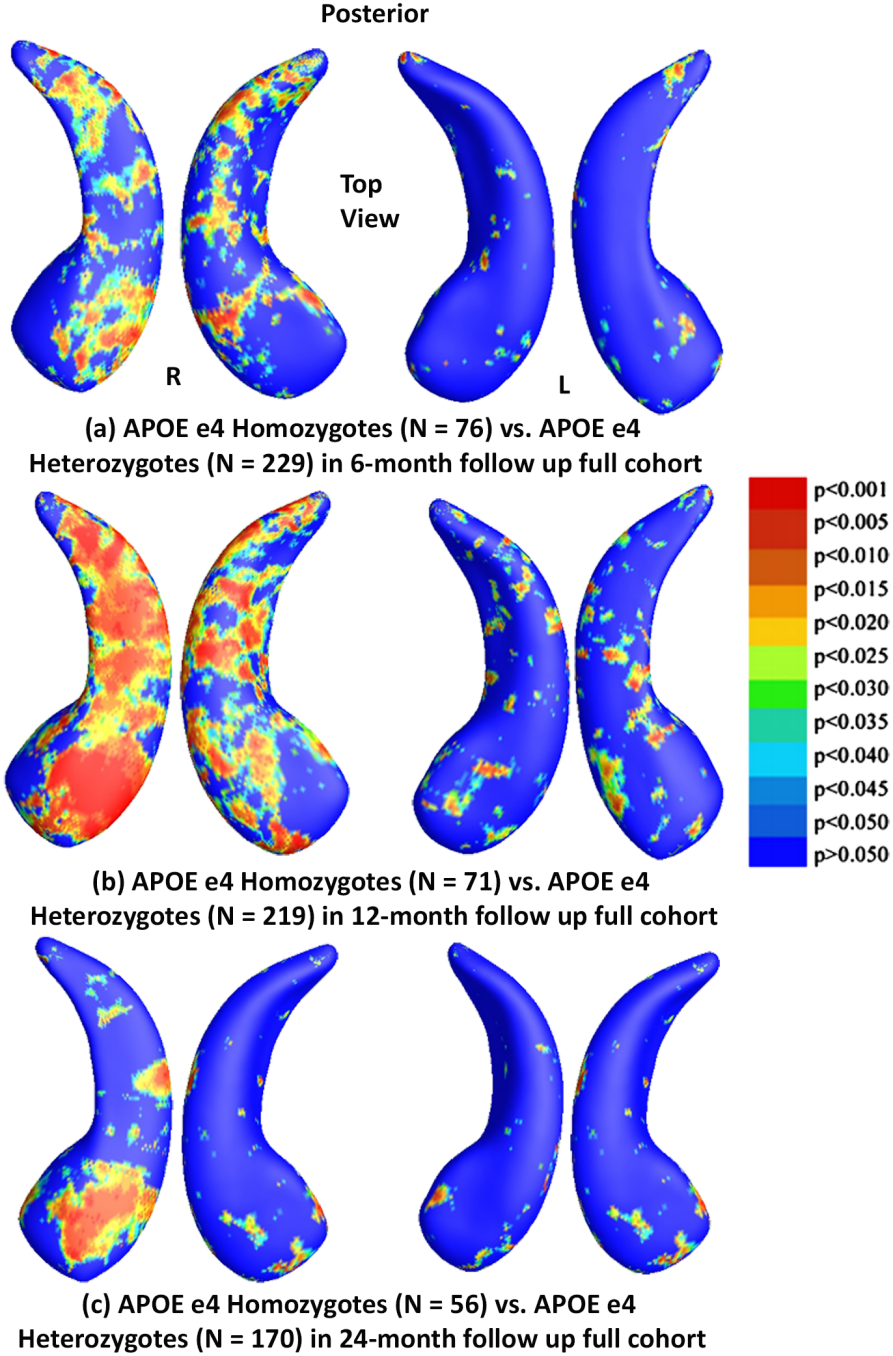
Shape Differences between Heterozygotes and Homozygotes in Full ADNI. (a), (b) and (c): Illustration of local shape differences (*p*-values) between the APOE e4 homozygotes (e4/e4) and heterozygotes (e3/e4) in the full ADNI cohorts at 6-months, 12-months and 24 months, respectively. Non-blue colours show vertices with statistical differences, at the nominal 0.05 level, uncorrected. The overall significance after multiple comparisons with permutation test is: (a) *p* < 0.0117, (b) *p* < 0.0024, (c) *p* < 0.0959.

To explore whether APOE e4 allele dose affects hippocampal surface morphometry and how this atrophy is related to normal aging, we studied hippocampal morphometry differences between persons homozygous for the APOE e4 allele and those heterozygous in three follow up cohorts. Fig 3 shows the statistical *p*-maps for these three different follow up time intervals, specifically, (a) for 6-month follow up (N = 305, 76 e4 homozygotes vs. 229 e4 heterozygots), (b) for 12-month (N = 290, 71 e4 homozygotes vs. 219 heterozygots), and (c) for 24-month (N = 226, 56 e4 homozygotes vs. 170 heterozygots). After correcting for multiple comparisons, the differences remained significant for the 6- and 12-month but not for the 24-month follow up cohort (*p*<0.0117 for 6-months, *p*<0.0024 for 12-months, *p*<0.0959 for 24-months).

**Fig 4:**
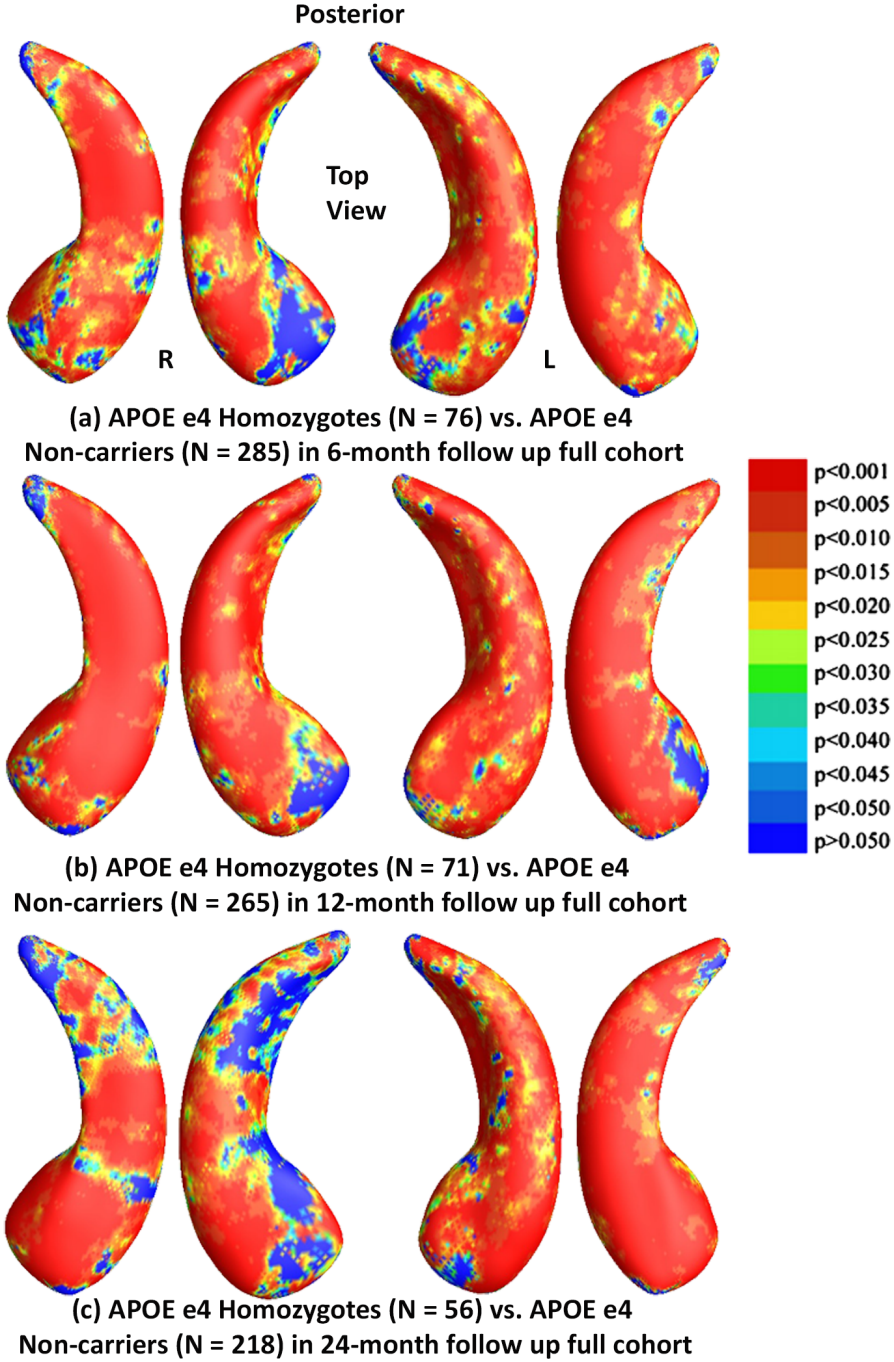
Shape Differences between Non-carriers and Homozygotes in Full ADNI. (a), (b) and (c): Illustration of local shape differences (*p*-values) between the APOE e4 homozygotes (e4/e4) and non-carriers (e3/e3) in the full ADNI cohorts at 6-months, 12-months and 24 months, respectively. Non-blue colours show vertices with statistical differences, at the nominal 0.05 level, uncorrected. The overall significance after multiple comparisons with permutation test is: (a) *p* < 0.0001, (b) *p* < 0.0001, (c) *p* < 0.0001.

**Fig 5:**
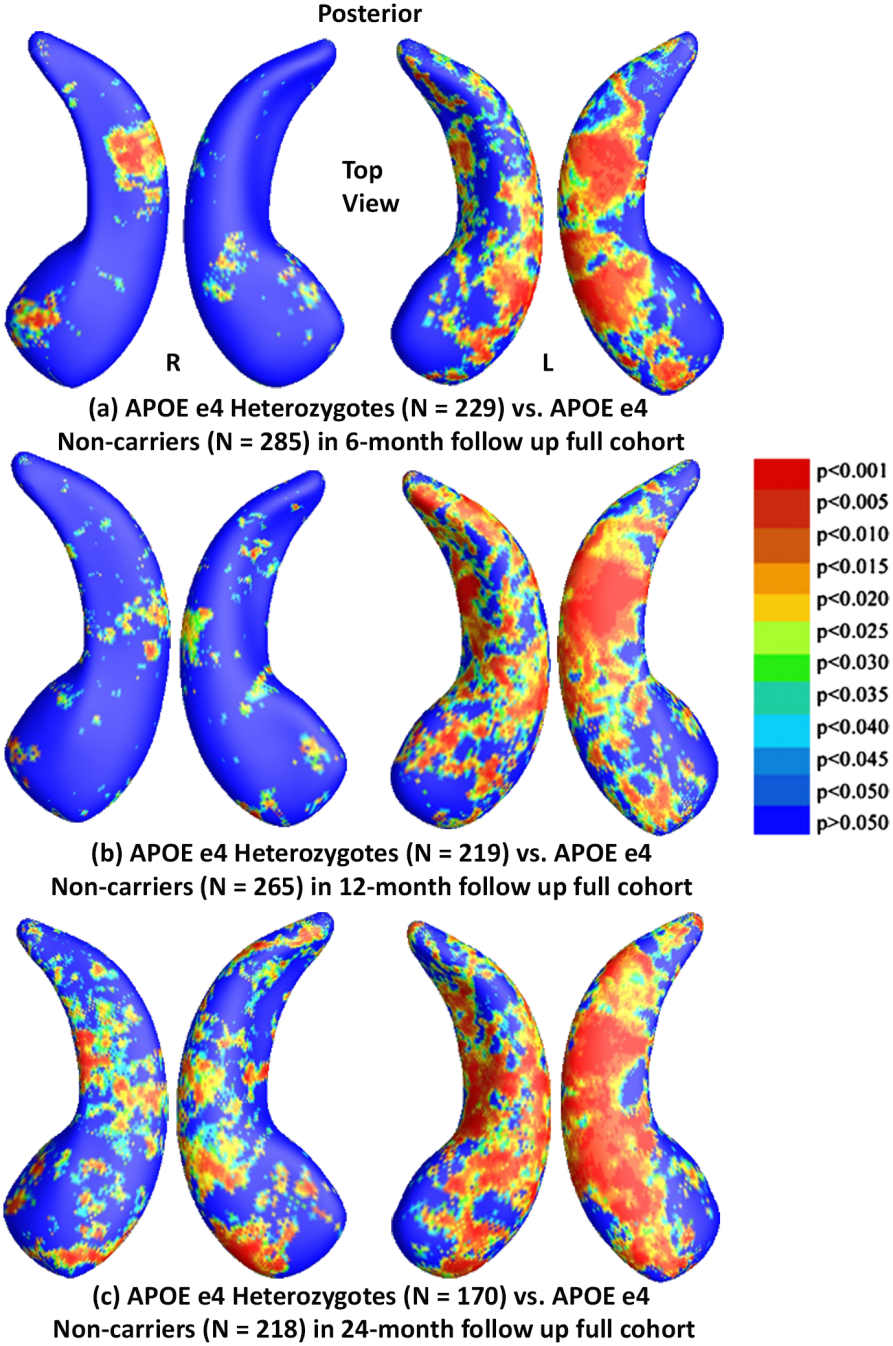
Shape Differences between Non-carriers and Heterozygotes in Full ADNI. (a), (b) and (c): Illustration of local shape differences (*p*-values) between the APOE e4 heterozygotes (e3/e4) and non-carriers (e3/e3) in the full ADNI cohorts at 6-months, 12-months and 24 months, respectively. Non-blue colours show vertices with statistical differences, at the nominal 0.05 level, uncorrected. The overall significance after multiple comparisons with permutation test is: (a) *p* < 0.0116, (b) *p* < 0.0039, (c) *p* < 0.0003.

We also studied hippocampal morphometry differences between APOE e4 non-carriers and carriers with different e4 dose. Figs 4 and 5 show how APOE e4 non-carriers differ in hippocampal shape from e4 homozygotes and heterozygotes, respectively. Fig 4 shows the statistical *p*-maps for comparisons between e4 homozygotes and non-carriers for (a) 6-month follow up (N = 361, 76 e4 homozygotes vs. 285 non-carriers), (b) for 12-month (N = 336, 71 e4 homozygotes vs. 265 non-carriers), and (c) for 24-month (N = 274, 56 e4 homozygotes vs. 170 non-carriers). After correcting for multiple comparisons, the differences remained significant for all three cohorts (*p*<0.0001 for all timepoints). Fig 5 shows the statistical *p*-maps for comparisons between e4 heterozygotes and non-carriers for (a) for 6-month follow up (N = 514, 229 e4 heterozygotes vs. 285 non-carriers), (b) for 12-month (N = 484, 219 e4 heterozygotes vs. 265 non-carriers), and (c) for 24-month (N = 388, 170 e4 heterozygotes vs. 218 non-carriers). After correcting for multiple comparisons, the differences remained significant for all three cohorts (*p*<0.0116 for 6-months, *p*<0.0039 for 12-months, *p*<0.0003 for 24-months).

### Results in the Non-demented ADNI Cohorts for Three Follow Up Intervals

**Fig 6:**
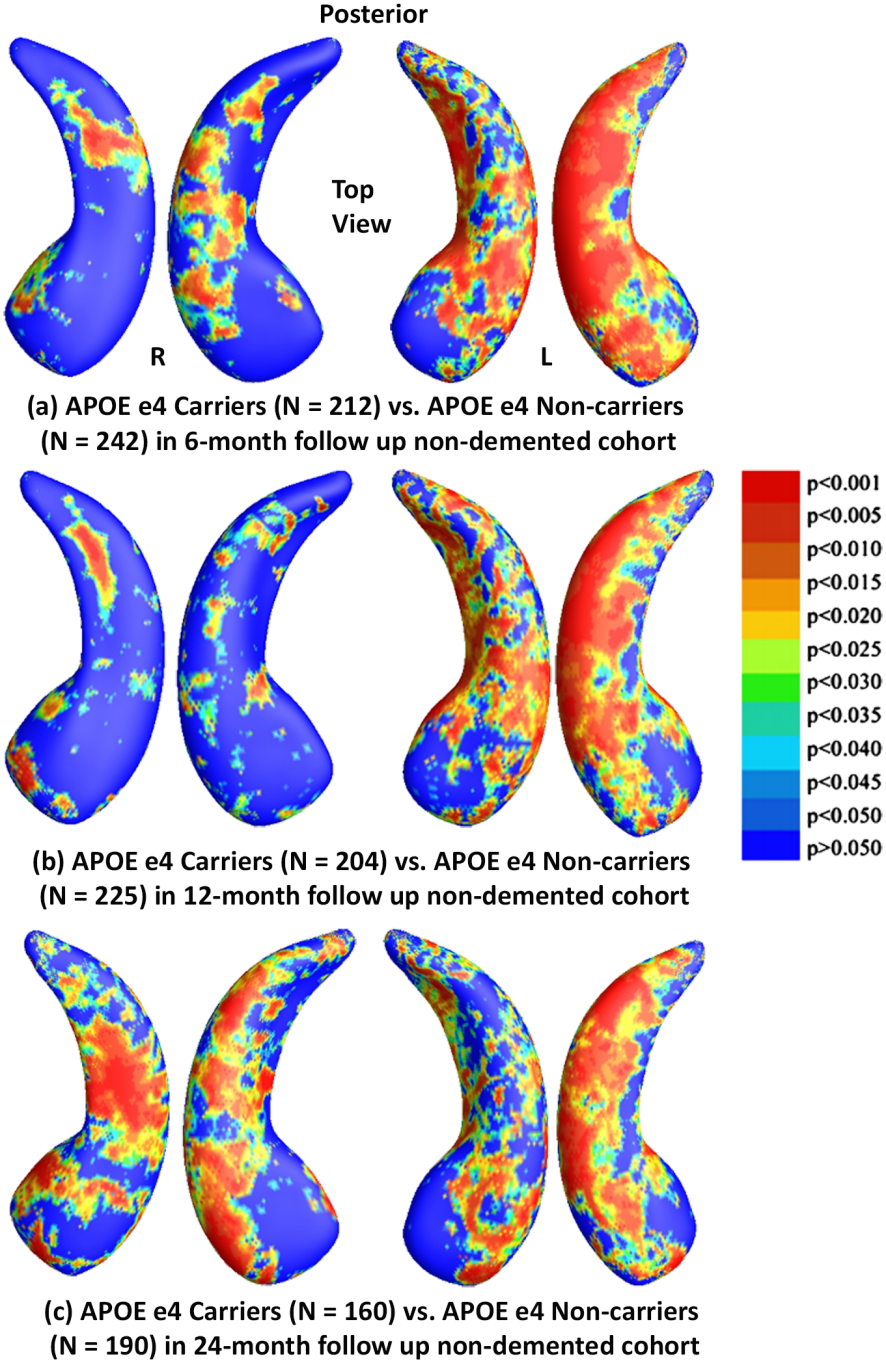
Shape Differences between Non-carriers and Carriers in Nondemented. (a), (b) and (c): Illustration of local shape differences (*p*-values) between the APOE e4 carriers **(e3/e4 and e4/e4)** and non-carriers **(e3/e3)** in the non-demented cohorts at 6-months, 12-months and 24-months, respectively. Non-blue colours show vertices with statistical differences, at the nominal 0.05 level, uncorrected. The overall significance after multiple comparisons with permutation test is: (a) *p* < 0.001, (b) *p* < 0.0005, (c) *p* < 0.0015.

We also conducted similar studies of APOE e4 genotype in three follow up non-demented ADNI cohorts. Fig 6 shows statistical *p*-maps for comparisons between e4 carriers and non-carriers in non-demented cohorts of three different follow up intervals, specifically, (a) for 6-month follow up (N = 454, 242 non-carriers vs. 212 e4 carriers), (b) for 12-month (N = 429, 225 non-carriers vs. 204 e4 carriers), and (c) for 24-month (N = 350, 190 non-carriers vs. 160 e4 carriers). Non-blue colours show vertices with statistical differences at the nominal 0.05 level, uncorrected for multiple comparisons. After correcting for multiple comparisons, the differences remained significant for all three cohorts (*p*<0.0010 for 6-months, *p*<0.0005 for 12-months, *p*<0.0015 for 24-months).

**Fig 7:**
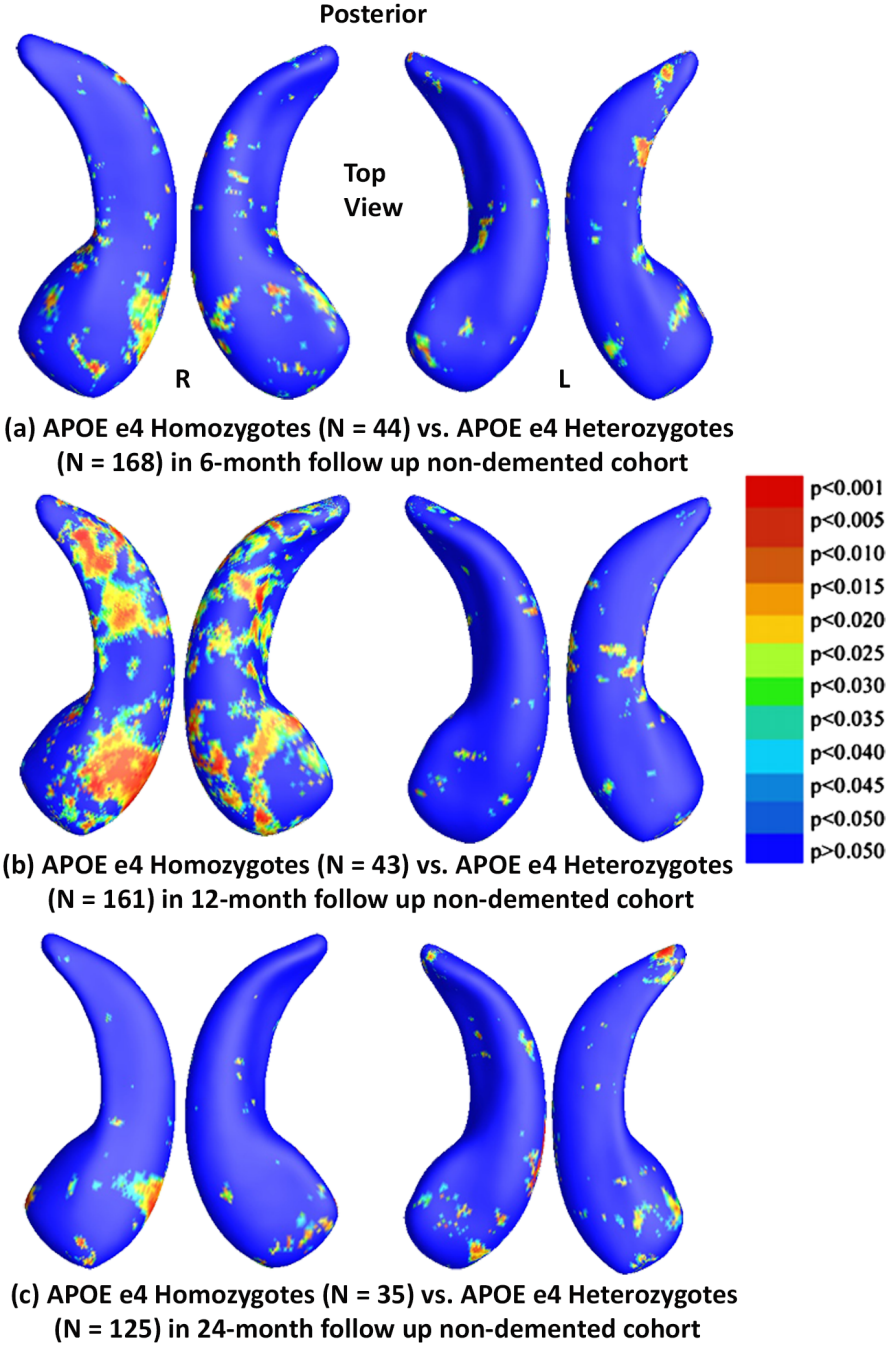
Shape Differences between Heterozygotes and Homozygotes in Nondemented. (a), (b) and (c): Illustration of local shape differences (*p*-values) between the APOE e4 homozygotes (e4/e4) and heterozygotes (e3/e4) in the non-demented cohorts at 6-months, 12-months and 24-months, respectively. Non-blue colours show vertices with statistical differences, at the nominal 0.05 level, uncorrected. The overall significance after multiple comparisons with permutation test is: (a) *p* < 0.1351, (b) *p* < 0.0204, (c) *p* < 0.187.

Similar to the full ADNI cohort studies, Fig 7 shows the statistical *p*-maps for comparisons between e4 homozygotes and heterozygotes in non-demented cohorts of three different follow up intervals, specifically, (a) for 6-month follow up (N = 212, 44 homozygotes vs. 168 heterozygotes), (b) for 12-month (N = 204, 43 homozygotes vs. 161 heterozygotes), and (c) for 24-month (N = 160, 35 homozygotes vs. 125 heterozygotes). After correcting for multiple comparisons, the differences only remained significant for 12-month follow up cohort (*p*<0.0204) and not for the other two cohorts (*p*<0.1351 for 6-months, *p*<0.1870 for 24-months).

**Fig 8:**
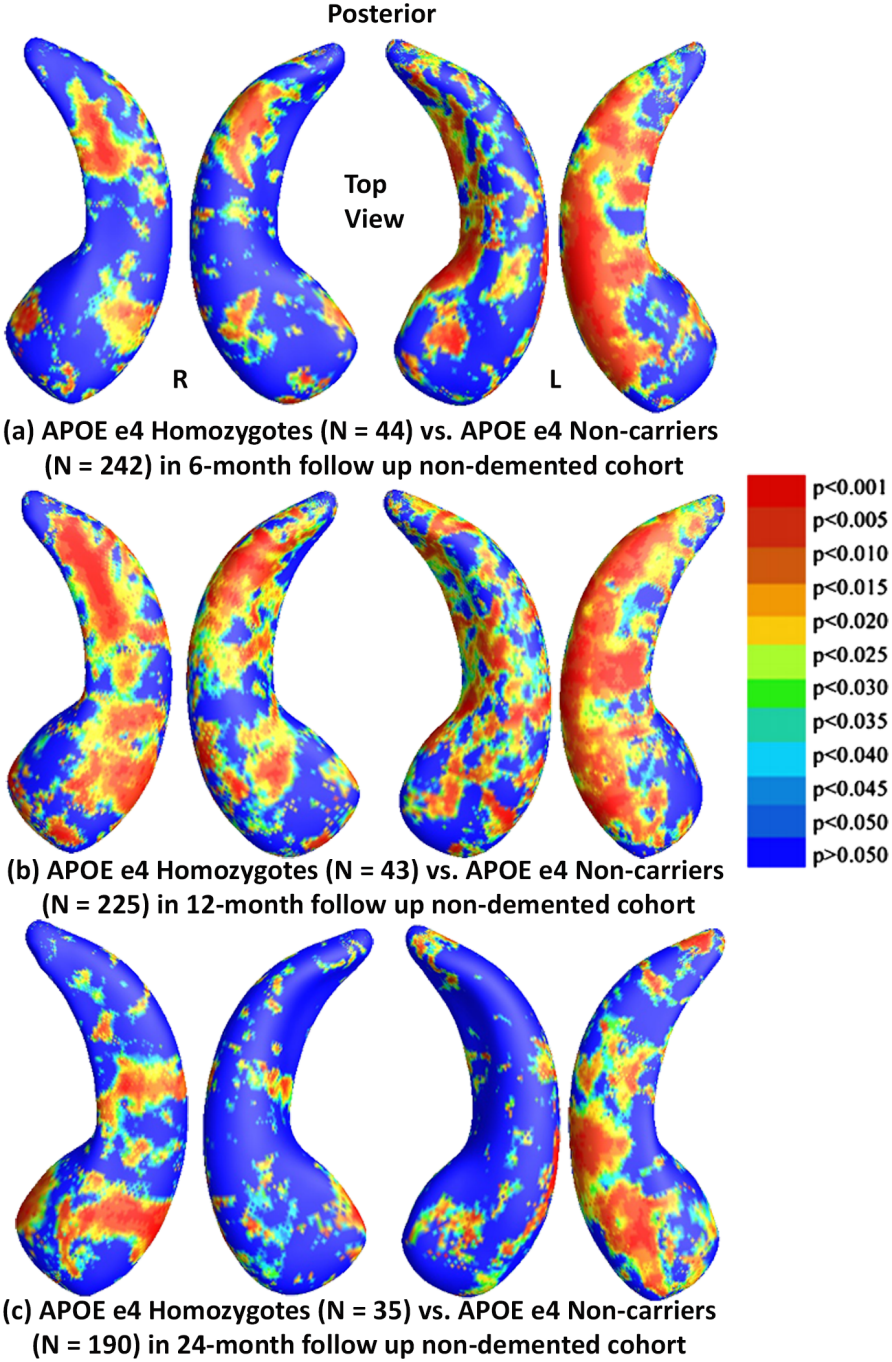
Shape Differences between Non-carriers and Homozygotes in Nondemented. (a), (b) and (c): Illustration of local shape differences (*p*-values) between the APOE e4 homozygotes **(e4/e4)** and non-carriers **(e3/e3)** in the non-demented cohorts at 6-months, 12-months and 24-months, respectively. Non-blue colours show vertices with statistical differences, at the nominal 0.05 level, uncorrected. The overall significance after multiple comparisons with permutation test is: (a) *p* < 0.0035, (b) *p* < 0.001, (c) *p* < 0.077.

**Fig 9:**
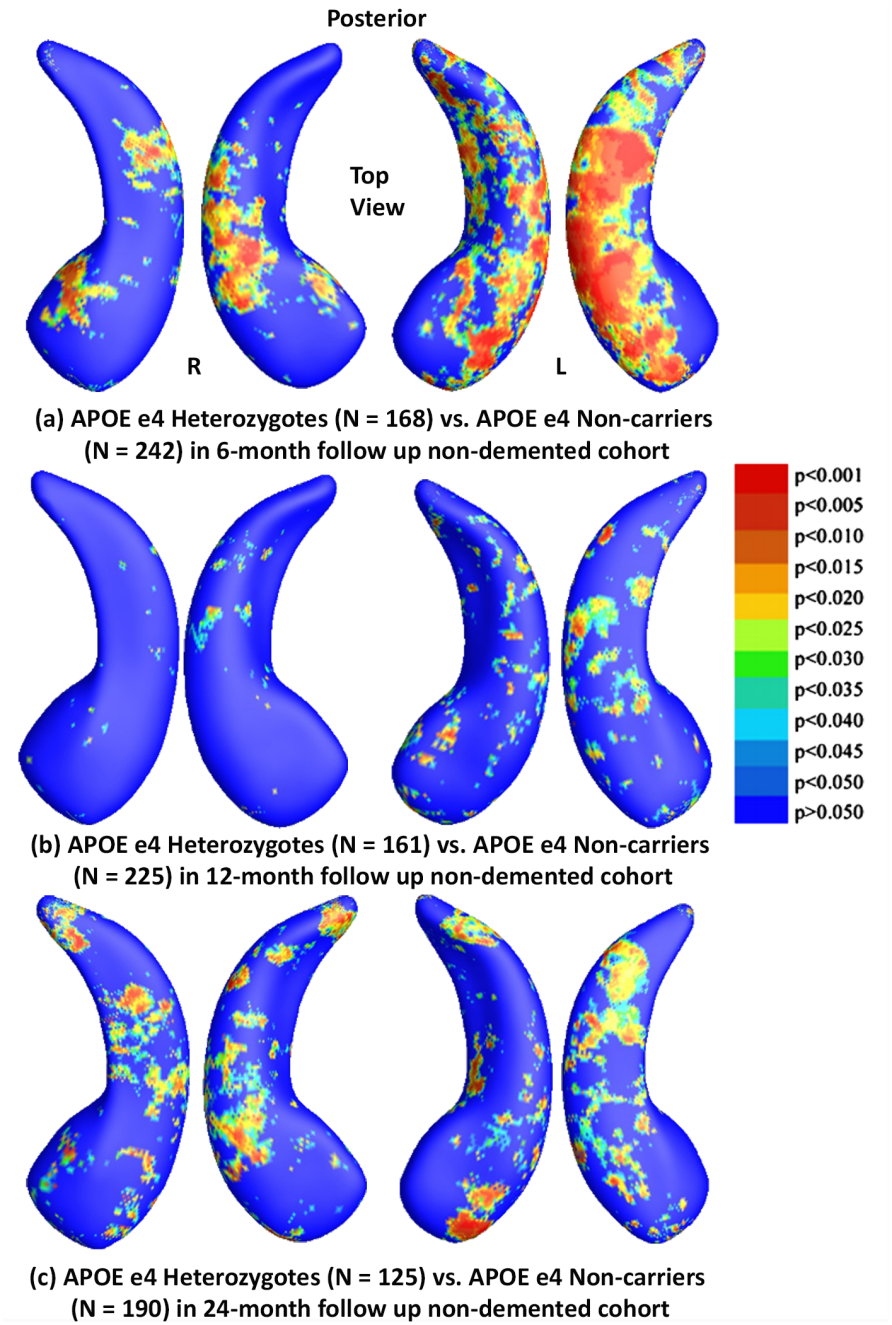
Shape Differences between Non-carriers and Heterozygotes in Nondemented. (a), (b) and (c): Illustration of local shape differences (*p*-values) between the APOE e4 heterozygotes **(e3/e4)** and non-carriers **(e3/e3)** in the non-demented cohorts at 6-months, 12-months and 24-months, respectively. Non-blue colours show vertices with statistical differences, at the nominal 0.05 level, uncorrected. The overall significance after multiple comparisons with permutation test is: (a) *p* < 0.0058, (b) *p* < 0.1191, (c) *p* < 0.011.

We also studied hippocampal morphometry differnces between APOE e4 non-carriers and carriers with different e4 dose in the non-demented cohorts. Fig 8 shows the statistical *p*-maps for comparisons between e4 homozygotes and non-carriers in three different follow up intervals, specifically, (a) for 6-month follow up (N = 286, 44 homozygotes vs. 242 non-carriers), (b) for 12-month (N = 268, 43 homozygotes vs. 225 non-carriers), and (c) for 24-month (N = 225, 35 homozygotes vs. 190 non-carriers). After correcting for multiple comparisons, the differences remained significant for 6- and 12-month follow up cohorts (*p*<0.0035 for 6-months and *p*<0.0010 for 12-months) but not for 24-month cohorts (*p*<0.0770 for 24-months Fig 9). shows the statistical *p*-maps for comparisons between e4 heterozygotes and e4 non-carriers, specifically, (a) for 6-month follow up (N = 410, 168 heterozygotes vs. 242 non-carriers), (b) for 12-month (N = 386, 161 homozygotes vs. 225 non-carriers), and **(c)** for 24-month (N = 315, 125 heterozygotes vs. 190 non-carriers). After correcting for multiple comparisons, the differences remained significant for 6- and 24-month follow up cohorts (*p*<0.0058 for 6-months and *p*<0.0110 for 24-months) but not for 12-month follow up cohorts (*p*<0.1191 for 12-months).

### Cumulative Distribution Functions of the *p*-values in the Statistical *p*-maps

**Fig 10:**
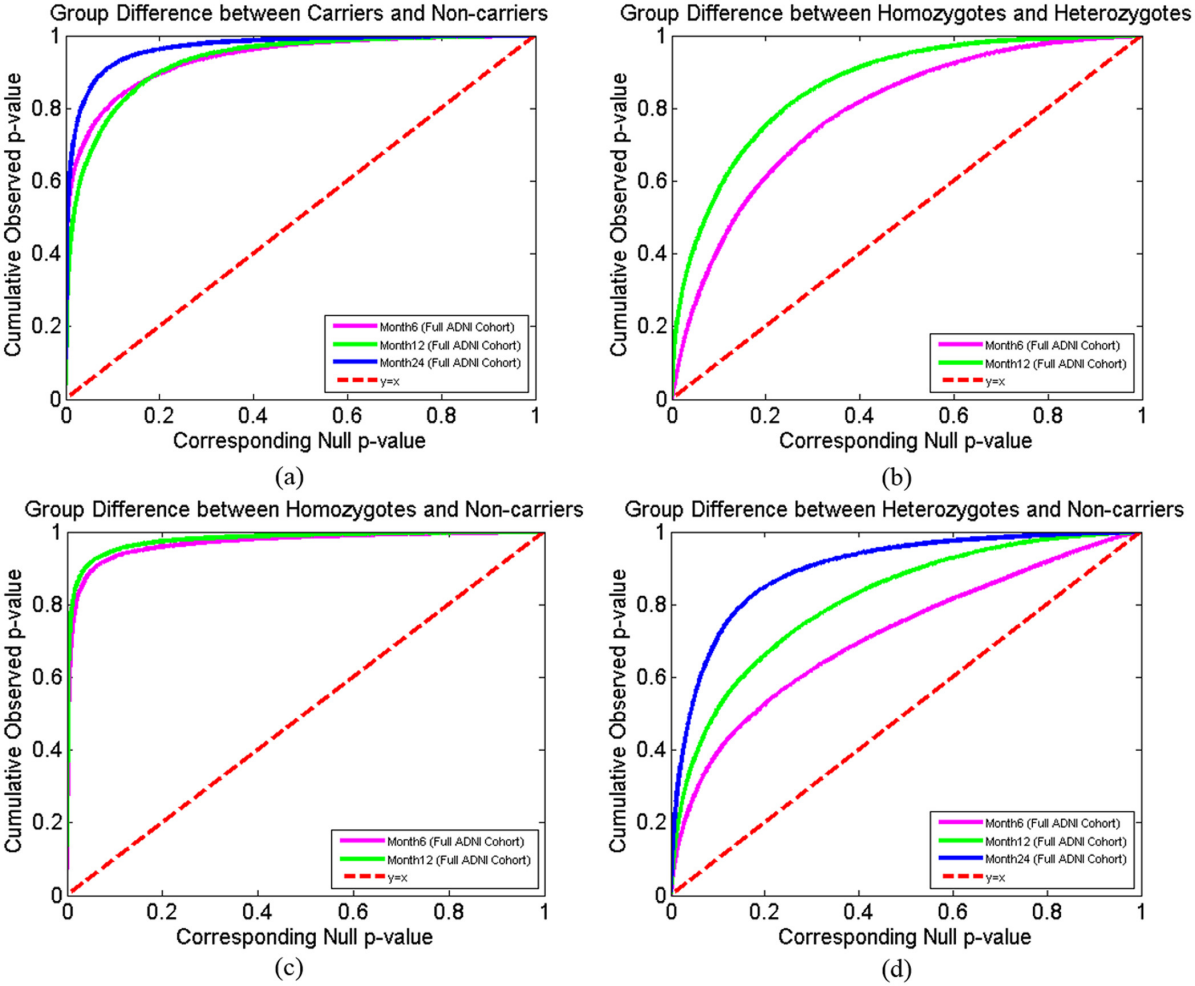
Cumulative Distribution Function Plots Comparison. Cumulative Distribution Function (CDF) plots comparison, between: (a). APOE e4 carriers **(e3/e4 and e4/e4)** and non-carriers **(e3/e3)**, (b). APOE e4 homozygotes **(e4/e4)** and heterozygotes **(e3/e4)**, (c). APOE e4 homozygotes **(e4/e4)** and non-carriers **(e3/e3)**, (d). APOE e4 heterozygotes **(e3/e4)** and non-carriers **(e3/e3)** in the full ADNI cohorts. The results demonstrate the accelarated hippocampal atrophy in the longitudinal study.

In Fig 10, we created a set of cumulative distribution functions (CDF) of the *p*-values observed in four group difference experiments in the full ADNI cohort. We chose those experimental results that passed the permutation based multiple comparison tests (i.e., after correcting for multiple comparisons, *p*<0.05). Since there are too few homozygote samples in the 24-month follow up cohort (56 subjects in the full ADNI cohort), we also excluded the homozygote related CDFs from the 24-month follow up cohort. The CDFs of *p*-values are plotted against the corresponding *p*-value that would be expected, under the null hypothesis of no group difference, for all above experiments shown in Fig 10. For null distributions, the cumulative distribution of *p*-values is expected to fall approximately along the dotted line. Large deviations from that curve are associated with significant signal, and greater effect sizes represented by larger deviations. The theory of false discovery rates (FDR) [76] gives formulae for thresholds that tend to control false positives at a known rate. This protocol was adopted in several of our prior papers [25, 40, 44, 46, 47, 55, 77] as an empirical standard to compare effects in group difference analysis. The deviation of the statistics from the null distribution generally increased longitudinally from 6-month, to 12-month and 24-month follow up data in the full ADNI cohort. It shows that the continually increasing differences in atrophy between APOE e4 carriers and non-carriers (Fig 10(a)), APOE e4 heterozygotes and homozygotes (Fig 10(b)), APOE e4 homozygotes and non-carriers (Fig 10(c)), and APOE e4 heterozygotes and non-carriers (Fig 10(d)).

## Discussion

Our analyses of the full ADNI cohort revealed significant differences between APOE e4 carriers and non-carriers in all three follow up cohorts (Fig 2), between e4 homozygotes and heterozygotes in the 6- and 12-month follow up cohorts (Fig 3), between e4 homozygotes and non-carriers in all three follow up cohorts (Fig 4), and between e4 heterozygotes and non-carriers in all three follow up cohorts (Fig 5). Also, from the CDFs shown in Fig 10, there is a trend for group differences to generally become sharper over time. These results are consistent with our observations in the baseline cohort [25] and another prior work [78] in a relatively small dataset, showing a clear relationship between APOE genotype and hippocampal atrophy in the full ADNI cohort at all three follow up time intervals. In contrast, some investigators, e.g. [79–83], detected no APOE e4 gene dose effect on hippocampal atrophy. In the full ADNI cohort, a relatively large imaging cohort (N = 1925), we found that the APOE e4 dose was associated with greater hippocampal deformation (i.e., the CDF curves in Fig 10(c) are much steeper than those in Fig 10(d)). Although more rigorous statistical comparisons are necessary, from the *p*-maps and CDF plots, we can observe the trend that in these groups shown in Fig 10(c) and 10(d), APOE e4 homozygotes appear to differ more from non-carriers than do e4 heterozygotes. To our knowledge, this is the first study to apply a surface-based approach to evaluate longitudinal APOE e4 gene dose effects on hippocampal morphometry. Our findings confirm and extend our observation that APOE e4 gene dose correlates with the severity of hippocampal deformation, and support the use of MRI hippocampal morphometry as a valid imaging biomarker to track AD progression.

Findings in the non-demented subset were consistent with our previously reported baseline findings [25]. With few exceptions (homozygotes vs heterozygotes in 6- and 24-month follow up cohorts, heterozygotes and non-carriers in the 12-month follow up cohort and homozygotes and non-carriers in the 24-month follow up cohort), we found significant differences in APOE subgroups in all comparisons in each follow up cohort. However, the effects of e4 homozygosity on regional patterns of hippocampal morphometry at 24-months did not pass the permutation tests when compared to heterozygotes or non-carriers, probably reflecting insufficient statistical power, as sample sizes were much smaller (N = 35) than other subject numbers. In our prior work [25], we did not detect statistically significant differences between homozygotes and heterozygotes at baseline in the non-demented cohort, but we now find increasing differences between non-demented homozygotes and heterozygotes in the 12-month follow up cohort, as well as between homozygotes and non-carriers in both the 6- and 12-month follow up cohorts, supporting our hypothesis that there is an e4 gene dose effect for hippocampal deformation in the non-demented population.

Previous investigators [80, 84–86] reported greater atrophy of the right hippocampus when comparing e4 heterozygotes and homozygotes with non-carriers. In contrast, our results suggest e4 carriers in general, as well as heterozygous and homozygous subgroups have greater deformity of the left hippocampus compared to non-carriers. By contrast, differences between the e4 heterozygous and homozygous subgroups were greater on the right side than on the left. Our results are consistent with some prior work [23, 25, 78]. The reason for this laterality effect is unclear, but may suggest that the APOE e4 dose effects start from the left side and subsequently extend to the right.

There is an ever growing variety of methods for examining the structure and function of the hippocampus via *in vivo* MR images [87, 88]. Some examine the subfields of the hippocampal formation and subregions of the parahippocampal gyrus [89–94], which segment hippocampus into different regions and analyze the volume and shape changes in these subfields. These methods compute volumetric image registration between template and individual subjects and translate and visualize the deformation of surfaces. Surface-based hippocampal shape analyses rely primarily on two components. First, they build an appropriate representation and correspondence between hippocampal shapes. Second, they carry out group analysis within this common domain. Surface parameterization methods [54, 95, 96] create a canonical space to match hippocampal surfaces. When the canonical space is a sphere, approaches based on spherical harmonic functions (SPHARM) [97–99] use coefficients of the harmonic expansion to infer shape differences between patient groups and controls. Another group of methods aims to build dense correspondence between surfaces [40, 100, 101]. For example, the Large Deformation Diffeomorphic Metric Mapping (LDDMM) [102] has been used to deform labeled anatomical templates of the hippocampus onto new images, using a combination of manual landmarking of points on the hippocampus and 3D fluid image registration [100, 101, 103]. Other dense mapping methods register hippocampal surfaces with surface geometric features [24, 35, 46, 52, 104, 105]. For group difference analysis, some groups have used a single low dimensional feature vector [106–108], or other detailed local geometric features such as medial distance [35], the LDDMM metric [109], and tensor-based morphometry [40] for detailed statistical shape analysis. This type of method benefits from high resolution information in the hippocampal surface representation and efficient numerical solutions to register and analyze surface deformations across subjects.

As noted in Shi, et al. [25], our current work has two main caveats. First, the ADNI participants are generally elderly, so they may not ideally represent patient populations in clinical prevention trials. Still, our current findings support the genetic influence of APOE genotype in non-demented cohorts. Second, we excluded APOE e2 carriers from our current study for a more focused study. We expect to conduct a thorough study on APOE e2 effects in our future research.

In conclusion, by applying our novel hippocampal morphometry system in the longitudinal ADNI datasets, we replicated the influence of APOE genotype on hippocampal morphometry observed at baseline [25], and demonstrated strong APOE e4 gene dose effects in the 6-month and 12-month follow up cohorts. In the future, we will continue developing novel imaging shape analysis systems to better detect genetic influences on the brain. We plan to apply this framework together with our ventricular morphometry system [40] and cortical thickness estimation system [77] in cognitively normal subjects to help detect preclinical AD [110, 111].
